# Identification of pharmacological agents that induce HMGB1 release

**DOI:** 10.1038/s41598-017-14848-1

**Published:** 2017-11-02

**Authors:** Peng Liu, Liwei Zhao, Friedemann Loos, Kristina Iribarren, Sylvie Lachkar, Heng Zhou, Lígia C. Gomes-da-Silva, Guo Chen, Lucillia Bezu, Gaelle Boncompain, Franck Perez, Laurence Zitvogel, Oliver Kepp, Guido Kroemer

**Affiliations:** 1 Faculty of Medicine, University of Paris Sud, Kremlin-Bicetre, France; 2 Cell Biology and Metabolomics Platforms, Gustave Roussy Cancer Campus, Villejuif, France; 3 Equipe 11 labellisée Ligue Nationale contre le Cancer, Centre de Recherche des Cordeliers, Paris, France; 4 Institut National de la Santé et de la Recherche Médicale (INSERM), UMR1138, Equipe labellisée Ligue Nationale Contre le Cancer, Paris, France; 5 Université Paris Descartes, Sorbonne Paris Cité, Paris, France; 6 Université Pierre et Marie Curie, Paris, France; 7 Institut Curie, PSL Research University, CNRS UMR144 Paris, France; 8 Institut de Cancérologie Gustave Roussy Cancer Campus (GRCC), Villejuif, France; 9 INSERM, U1015 Villejuif, France; 10 Center of Clinical Investigations, CIC1428 Villejuif, France; 11 Pôle de Biologie, Hôpital Européen Georges Pompidou, AP-HP, Paris, France; 12 Department of Women’s and Children’s Health, Karolinska University Hospital, Stockholm, Sweden

## Abstract

The translocation of the protein high mobility group box 1 (HMGB1) from the nucleus to the cytoplasm and its secretion or passive release through the permeabilized plasma membrane, constitutes a major cellular danger signal. Extracellular HMGB1 can interact with pattern recognition receptors to stimulate pro-inflammatory and immunostimulatory pathways. Here, we developed a screening assay to identify pharmacological agents endowed with HMGB1 releasing properties. For this, we took advantage of the “retention using selective hooks” (RUSH) system in which a streptavidin-NLS3 fusion protein was used as a nuclear hook to sequestrate streptavidin-binding peptide (SBP) fused with HMGB1 and green fluorescent protein (GFP). When combined with biotin, which competitively disrupts the interaction between streptavidin-NLS3 and HMGB1-SBP-GFP, immunogenic cell death (ICD) inducers such as anthracyclines were able to cause the nucleo-cytoplasmic translocation of HMGB1-SBP-GFP. This system, was used in a high-content screening (HCS) campaign for the identification of HMGB1 releasing agents. Hits fell into three functional categories: known ICD inducers, microtubule inhibitors and epigenetic modifiers. These agents induced ICD through a panoply of distinct mechanisms. Their effective action was confirmed by multiple methods monitoring nuclear, cytoplasmic and extracellular HMGB1 pools, both in cultured human or murine cells, as well as in mouse plasma.

## Introduction

High mobility group box 1 (HMGB1) is a protein that is normally localized in the nucleus, where it is the most abundant non-histone chromatin-binding protein. In contrast to histones, that are part of nucleosomes, the interaction of HMGB1 with chromatin is rather loose, meaning that HMGB1 can exit from nuclei to the cytoplasm. HMGB1 can be released from the cells by non-canonical secretion pathways or passively liberated through the permeabilized plasma membrane of dead cells. Under homeostatic conditions HMGB1 bidirectionally shuttles between the cytoplasm and the nucleus, yet predominantly resides in the nucleus due to two nuclear localization and two non-classical nuclear export signals^1,2^. A fraction of the nuclear pool of HMGB1 is continuously exported through the exportin chromosome region maintenance 1 (CRM1) system and re-imported due to its nuclear localization sequence (NLS) motifs^3^. JAK-STAT-dependent lysine hyperacetylation within the NLS sites blocks nuclear re-import and leads to a cytoplasmic aggregation of HMGB1^4^, as it occurs in monocytes responding to inflammatory signals including lipopolysaccharide (LPS) and tumor necrosis factor (TNF)^5^.

HMGB1 lacks signal peptides for classical endoplasmic reticulum and Golgi apparatus-dependent secretion. Thus, exocytosis via secretory lysosomes as well as caspase-1-dependent release downstream of inflammasome activation have been suggested as routes for HMGB1 release from activated monocytes and infected macrophages, respectively^6,7^. Nevertheless, the exact mechanism of extracellular release of HMGB1 during non-necrotic instances of cell death remains elusive.

As HMGB1 changes its subcellular localization, it radically modifies its function. In the nucleus, HMGB1 binds to chromatin and changes the architecture of the DNA, thereby enhancing transcription and replication from chromatin templates. Thus, HMGB1 can be considered as a transcriptional modifier^8^. Cytoplasmic HMGB1 has been shown to regulate mitophagy, in the extracellular space, however, HMGB1 serves as a danger-associated molecular pattern (DAMP) that either acts alone or complexed to other factors (DNA, RNA, bacterial lipopolysaccharide…) by binding to pattern recognition receptors including, but probably not limited to, toll-like receptor-2 (TLR2), toll-like receptor-4 (TLR4) and advanced glycosylation end-product specific receptor (AGER)^9–13^. Hence, HMGB1 contributes to danger signaling in a variety of contexts, thereby exerting pro-inflammatory and immunostimulatory effects.

One situation in which HMGB1 plays a major role is immunogenic cell death (ICD)^14–20^. Infectious pathogens or anticancer chemotherapeutics can induce ICD, thereby setting of an immune response against pathogen- or tumor-associated antigens. In this context, HMGB1 is released from dying and dead cells and interacts with TLR4 to stimulate the antigen-presenting function of maturing dendritic cells. Knockout of HMGB1 in cancer cells, its neutralization with specific antibodies or knockout of TLR4 in the host immune system, hence diminish the immune response and tumor growth-reducing effects of chemotherapy with anthracyclines and oxaliplatin in mouse models^16,21,22^. Moreover, loss of HMGB1 expression in malignant cells or loss-of-function alleles of TLR4 compromise the prognosis of patients with breast cancer undergoing adjuvant chemotherapy with anthracyclines or colorectal cancer treated with oxaliplatin-based chemotherapy^16,21,23–26^.

Given the immunological and oncological importance of nuclear HMGB1 exodus, we decided to identify pharmacological agents that stimulate nucleo-cytoplasmic HMGB1 translocation. Here, we report the design of a screening system for this purpose. Moreover, we enumerate several HMGB1 release inducers with confirmed *in vitro* and *in vivo* effects.

## Results and Discussion

### A screening system for measuring nuclear HMGB1 release

**Figure 1:**
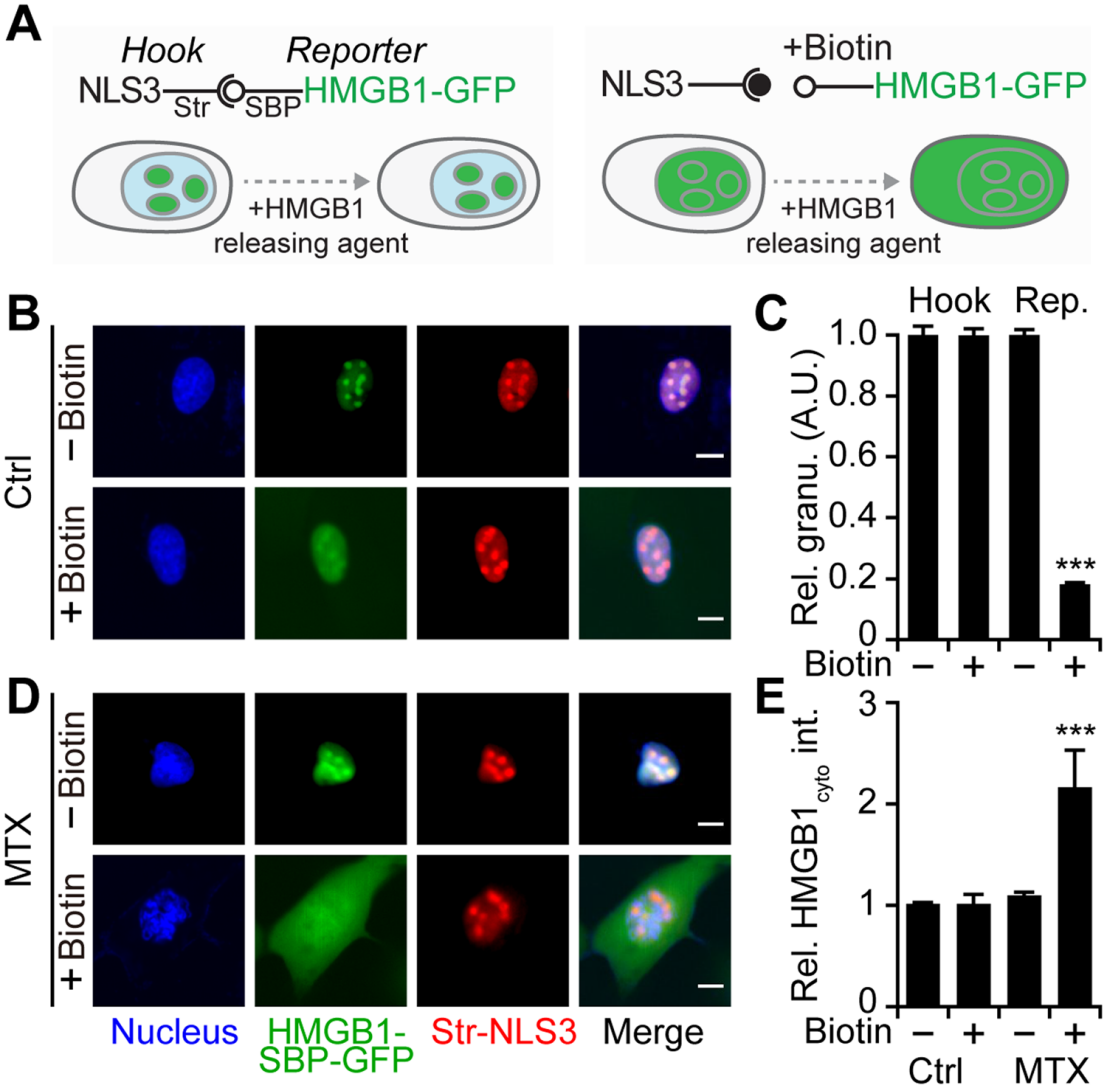
An optimized fluorescent biosensor–based cell line for the identification of HMGB1 releasing agents. (**A**) Scheme of the RUSH (Retention Using Selective Hooks)-HMGB1 cell assay. U2OS cells stably expressing Streptavidin-NLS3 (Str-NLS3) as a hook that localizes in the nuclear dots and can be detected with an anti-streptavidin antibody ((**B)** red color). In the absence of biotin, HMGB1-SBP-GFP is retained in the nucleus due to the streptavidin-SBP interaction and co-localizes with Str-NLS3. Upon biotin addition, the HMGB1-SBP-GFP reporter is released from the hook and diffuses in the nucleus, while the hook remains punctiform. Granularity of HMGB1-GFP and streptavidin-AF568 was quantified after the cells were exposed to biotin (**C**). (**D**) HMGB1 releasing agent mitoxantrone (MTX, 2 µM) induced the exodus of HMGB1 only in the presence of biotin. Cytoplasmic HMGB1 was quantified after the cells were exposed to MTX either in the absence or presence of biotin (**E**). Data are reported as means ± SEM at 24 h post treatment (n = 4; *P < 0.05, **P < 0.01, and ***P < 0.001, two-tailed Student’s t test, compared to control cells). Scale bar = 10 µm.

To measure HMGB1 release from the nucleus in an optimal fashion, we took advantage of the so called “retention using selective hooks” (RUSH) system (Fig. 1A)^27^. In this system, the protein of interest (here HMGB1) is fused to a streptavidin-binding peptide (SBP) as well as a green fluorescent protein (GFP) to facilitate monitoring of its subcellular localization by fluorescence videomicroscopy. Such a construct is stably expressed in cells (here human osteosarcoma U2OS cells) together with streptavidin that is targeted towards a specific subcellular compartment, here to the nucleus by means of three nuclear localization sequences (Str-NLS3) motif (Fig. S1). Driven by the interaction between SBP and streptavidin, the protein of interest together with its GFP tag is retained by the nuclear-targeted streptavidin protein, used as a hook. We observed that Str-NLS3 localized in punctiform structures within the nucleus together with the HMGB1-SBP-GFP fusion protein (Fig. 1B). Biotin has a subnanomolar affinity to streptavidin and can outcompete its binding to SBP, causing rapid release of the HMGB1-SBP-GFP fusion protein from its interaction with streptavidin-NLS3. However, HMGB1-SBP-GFP remained retained in the nucleus, where it was diffusely distributed, similarly to what is observed for endogenous HMGB1^21^. It is worth noting that the localization of the Str-NLS3 was not changed upon addition of biotin (Fig. 1C). The signal distribution changed when an agent that readily releases HMGB1 from the nucleus, namely the anthracycline mitoxantrone (MTX) was added to the cells. When MTX was combined with biotin, HMGB1-SBP-GFP appeared in the cytoplasm. However, MTX alone, without biotin, failed to cause such a redistribution of HMGB1-SBP-GFP (Fig. 1D,E). Fluorescence videomicroscopy allowed for real time observations of this process (Fig. S2, Videos S1 and S2). We reasoned that this kind of experimental design would introduce a marked degree of specificity into the system, meaning that non-specific destruction of the cells or fluorescence quenching would cause a loss of the nuclear HMGB1-SBP-GFP signal (Fig. S3) that would not depend on biotin addition. In contrast, specific inducers of HMGB1 release would cause the loss of the nuclear GFP signal only in the presence of biotin.

### Identification of FDA-approved drugs that release HMGB1 from the nucleus

**Figure 2:**
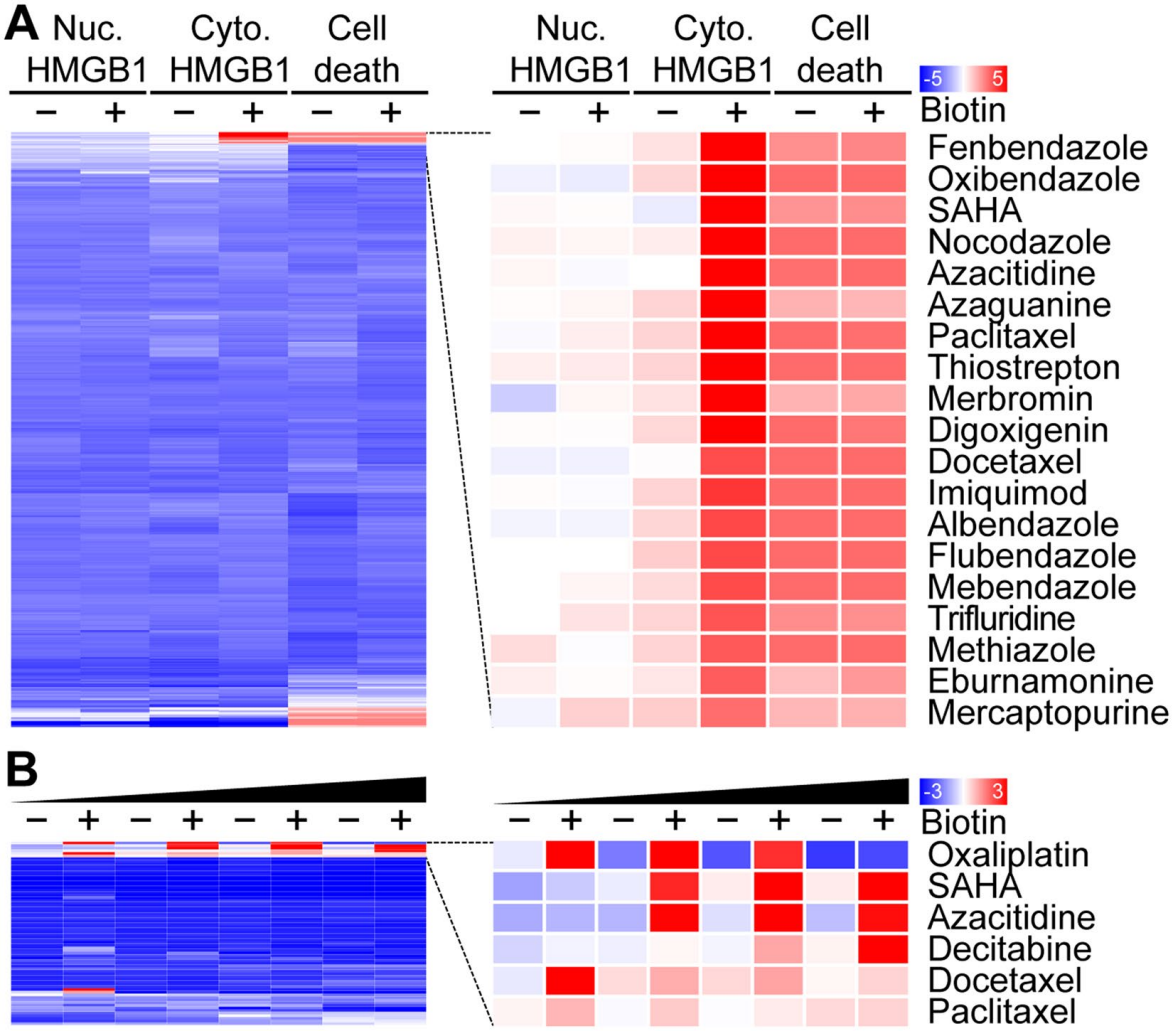
Identification of HMGB1 releasing agents from chemical libraries. (**A**) U2OS cells stably co-expressing streptavidin-NLS3 and HMGB1-SBP-GFP were seeded in 384-well plates either in the absence or in the presence of biotin before treatment with 1200 small molecules from the Prestwick chemical library (most of which are approved by FDA, EMA and other agencies) at a final concentration of 10 µM for 48 h. Following HMGB1-GFP fluorescence was quantified in the nucleus as well as the cytoplasm and, based on Hoechst 33342 staining, nuclear pyknosis was assessed as an indicator for cell death. Quantitative data were normalized by z-scoring (mean, n = 4) hierarchically clustered and depicted as heat map, with red and blue values indicating positive and negative effects, respectively. The top 20 compounds are shown. (**B**) U2OS cells stably co-expressing streptavidin-NLS3 and HMGB1-SBP-GFP were pre-incubated or not with biotin before treatment with a collection of FDA-approved anticancer agents at different concentrations for 48 h. Following cytoplasmic HMGB1-SBP-GFP fluorescence was quantified. Obtained results were hierarchically clustered (average linkage*, similarity metric using pearson distance*) and depicted as a heat map. Agents from the custom library have been used at the following concentrations azacitidine (1, 5, 10, 20 µM); paclitaxel (0.1, 0.5, 1, 2 µM); docetaxel, (0.1, 0.5, n 1, 2 µM); oxaliplatin (10, 50, 100, 200 µM); decitabine (1, 5, 10, 20 µM) and suberoylanilide hydroxamic acid (SAHA; 1, 5, 10, 20 µM).

**Figure 3:**
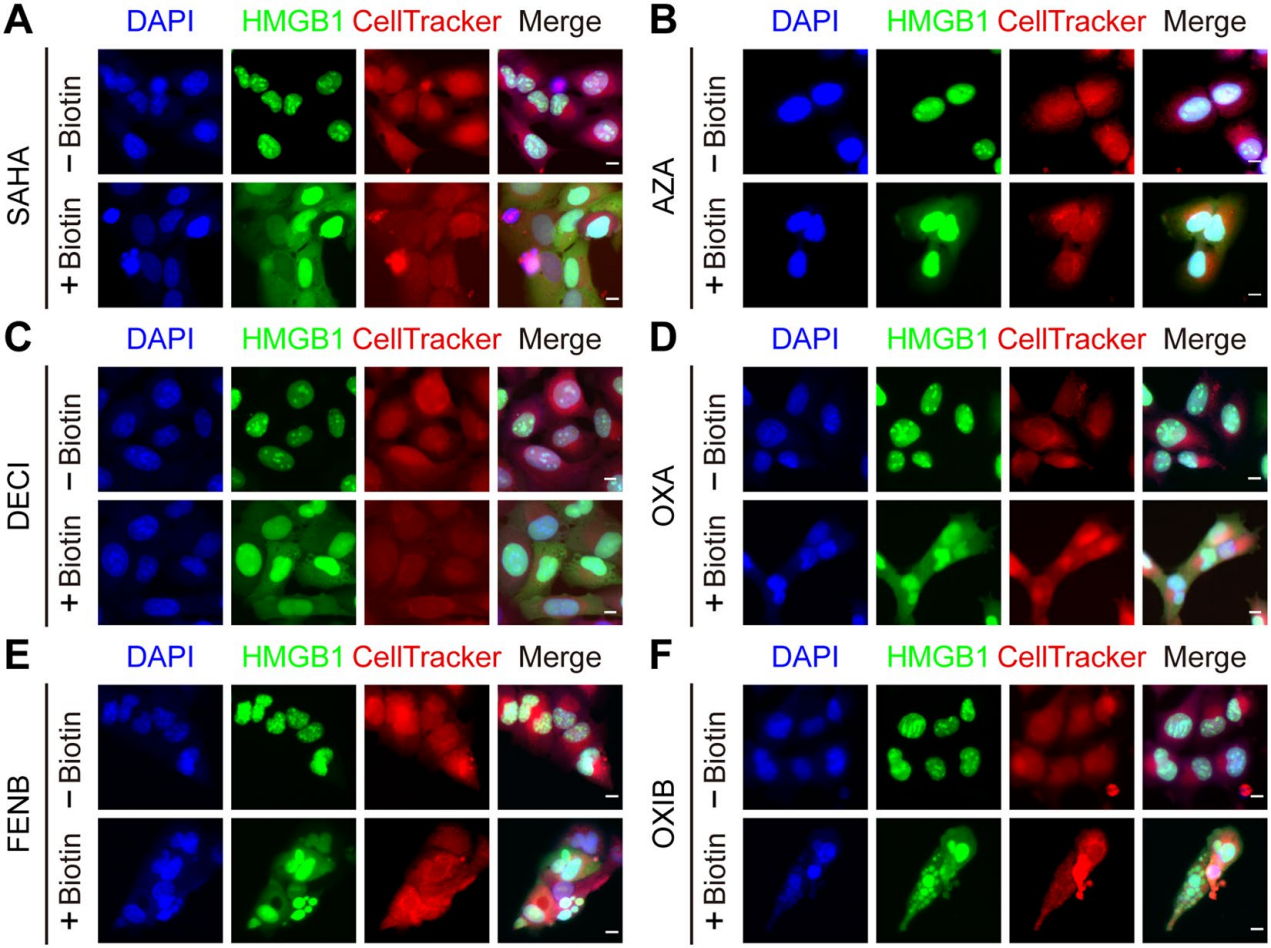
Nucleo-cytoplamic translocation of HMGB1-SBP-GFP. U2OS cells stably co-expressing Streptavidin-NLS3, HMGB1-SBP-GFP were pre-incubated or not with biotin before treatment with suberoylanilide hydroxamic acid (SAHA), fenbendazole (FENB) and oxibendazole (OXB), azacitidine (AZA), decitabine (DECI) all at 10 µM, and oxaliplatin (OXA) at 100 µM  for 48 h. Hoechst 33342 and CellTracker Orange CMTMR dye were used to visualize the nucleus and cytoplasmic region, respectively. The chemical staining allowed for the segmentation of nuclear and cytoplasmic areas by image analysis for subcellular HMGB1-GFP intensity using the MetaXpress software. Scale bar = 10 µm.

**Figure 4:**
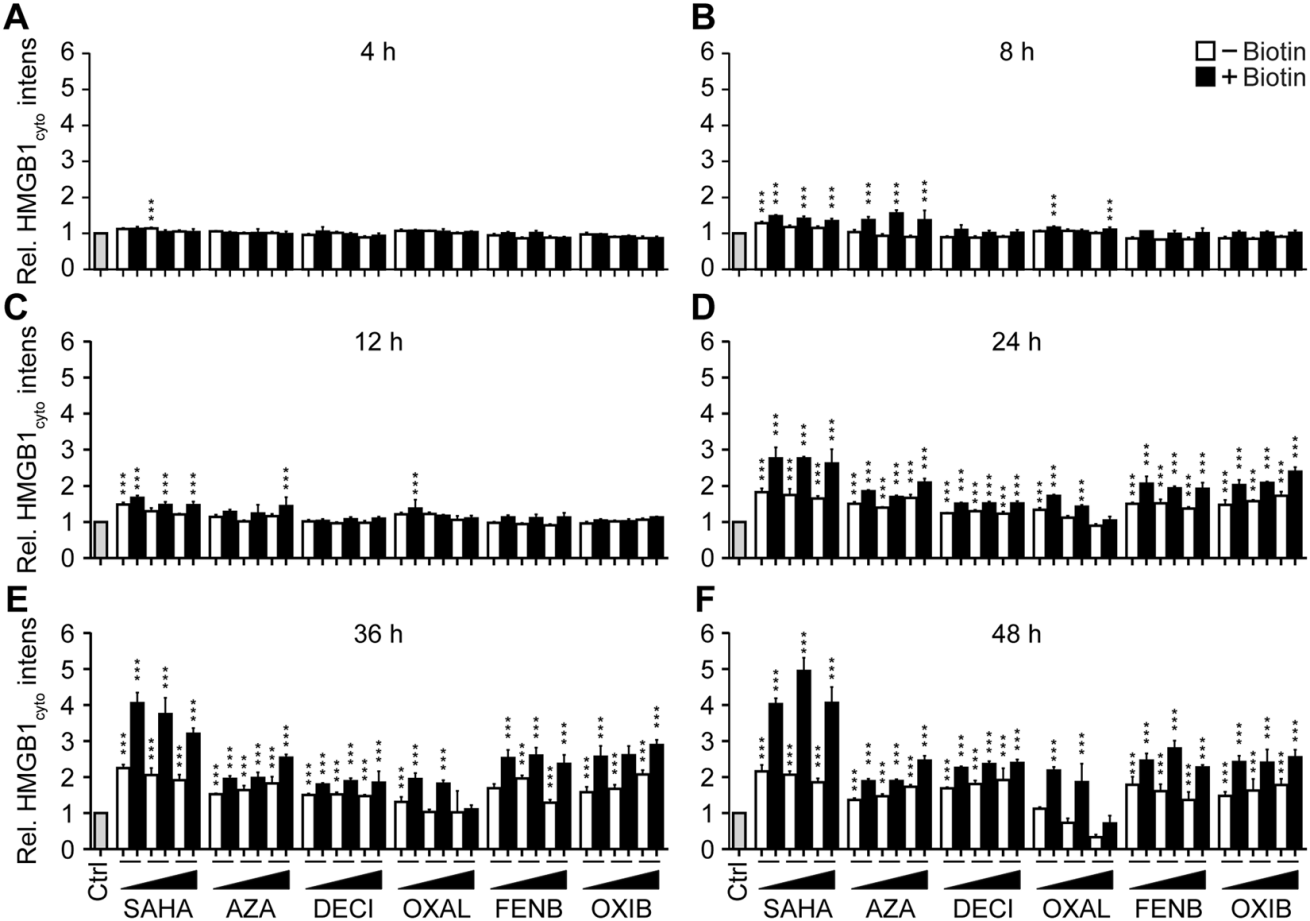
SAHA, Azacitidine, Decitabine, Oxaliplatin, Fenbendazole and Oxibendazole induce time/dose-dependent HMGB1 release. U2OS-SBP-HMGB1, Streptavidin-NLS3 co-expressing cells were pre-incubated or not with biotin before treatment with suberoylanilide hydroxamic acid (SAHA), fenbendazole (FENB) and oxibendazole (OXB) at 5, 10 and 20 µM, azacitidine (AZA) at 7.5, 15 and 30 µM, decitabine (DECI) at 10, 20 and 40 µM; and oxaliplatin (OXA) at 50, 100 and 200 µM for the indicated time points. Cells were then stained with Hoechst 33342 and CellTracker Orange CMTMR before fixation with 4% paraformaldehyde. Cytoplasmic GFP intensity was quantified and normalized to untreated controls. Data are shown as means ± SEM (n = 4; *P < 0.05, **P < 0.01, and ***P < 0.001, two-tailed Student’s t test, compared to control cells).

In the next step, we used the aforementioned system to screen the 1200 drugs contained in the Prestwick chemical library for their capacity to induce an increase in cytoplasmic HMGB1-SBP-GFP levels that would exclusively be detectable in the presence of biotin. Among the top-20 agents inducing this phenomenon, we found two epigenetic modifiers (azacitidine and suberoylanilide hydroxamic acid, SAHA), several microtubule inhibitors (docetaxel, paclitaxel and nocodazole) as well as several anthelmintic agent (albendazole, fenbendazole, flubendazole, mebendazole, oxibendazole) that exert their mode of action by binding to tubulin and thereby inhibit microtubule formation (Fig. 2A). We performed an additional screen on the collection of all small molecules that are FDA-approved anticancer agents and confirmed the capacity of epigenetic modifiers (azacitidine, decitabine, SAHA) and inhibitors of microtublular dynamics (docetaxel, paclitaxel) to efficiently induce the cytoplasmic accumulation of HMGB1-SBP-GFP levels (Fig. 2B). As expected, immunogenic cell death inducers such as the cardiac glycoside digoxigenin and the platinum-based antineoplastic oxaliplatin also induced the release of HMGB1-SBP-GFP from the nucleus^21,28^. Representative images of the biotin-dependent HMGB1-SBP-GFP-releasing activity of SAHA, azacitidine, decitabine, oxaliplatin, fenbendazole and oxibendazole are shown in Fig. 3. The dose and time dependency of such effects are documented in Fig. 4.

### *In vitro* and *in vivo* validation of HMGB1 releasing effects

**Figure 5:**
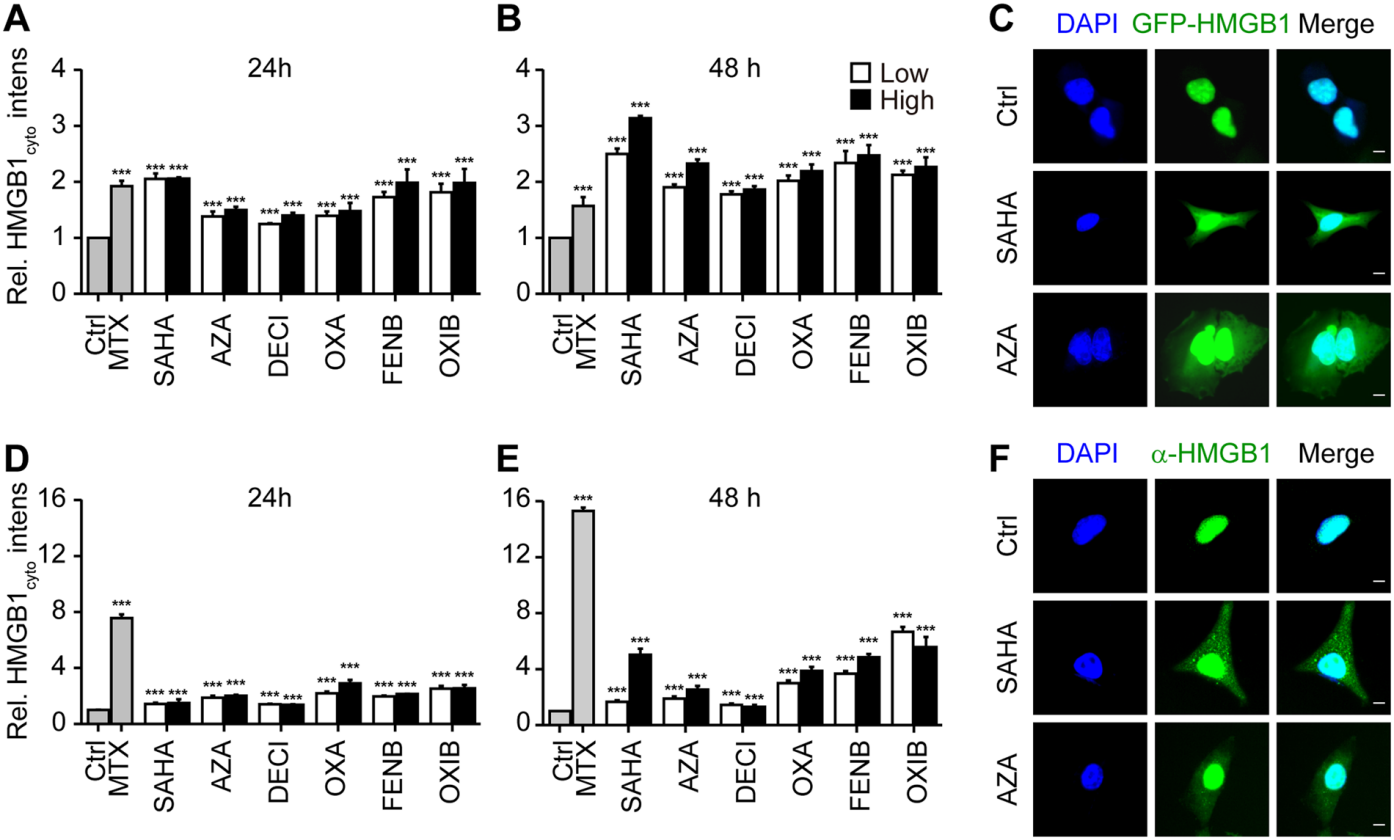
SAHA, azacitidine, decitabine, oxaliplatin, fenbendazole and oxibendazole induce HMGB1 release in regular biosensor cells and wild-type cells. U2OS cells stably expressing a GFP-HMGB1 fusion (**A–C**) or wild-type U2OS cells (**D–E**) were maintained in control conditions (Ctrl); mitoxantrone (MTX) at 2 µM; suberoylanilide hydroxamic acid (SAHA), fenbendazole (FENB) and oxibendazole (OXB) at 5 (l), 10 (h) µM; azacitidine (AZA) at 15 (l), 30 (h) µM; decitabine (DECI) at 20 (l), 40 (h) µM; and oxaliplatin (OXA) at 50 (l), 100 (h) µM for 24 h or 48 h, followed by assessment of cytoplasmic GFP-HMGB1 fluorescence intensity (**A**,**B**) or endogenous HMGB1 level after immunostaining with an anti-HMGB1 antibody and Alexa-fluor 488 conjugated 2^nd^ antibody and quantification of relative enrichment in the cytosol (**D**,**E**). Representative images of biosensor cell line (**C**) and immunofluorescence (**F**) are reported (scale bar = 10 µm). Data are shown as means ± SEM (n = 4; *P < 0.05, **P < 0.01, and ***P < 0.001, two-tailed Student’s t test, compared to control cells).

**Figure 6:**
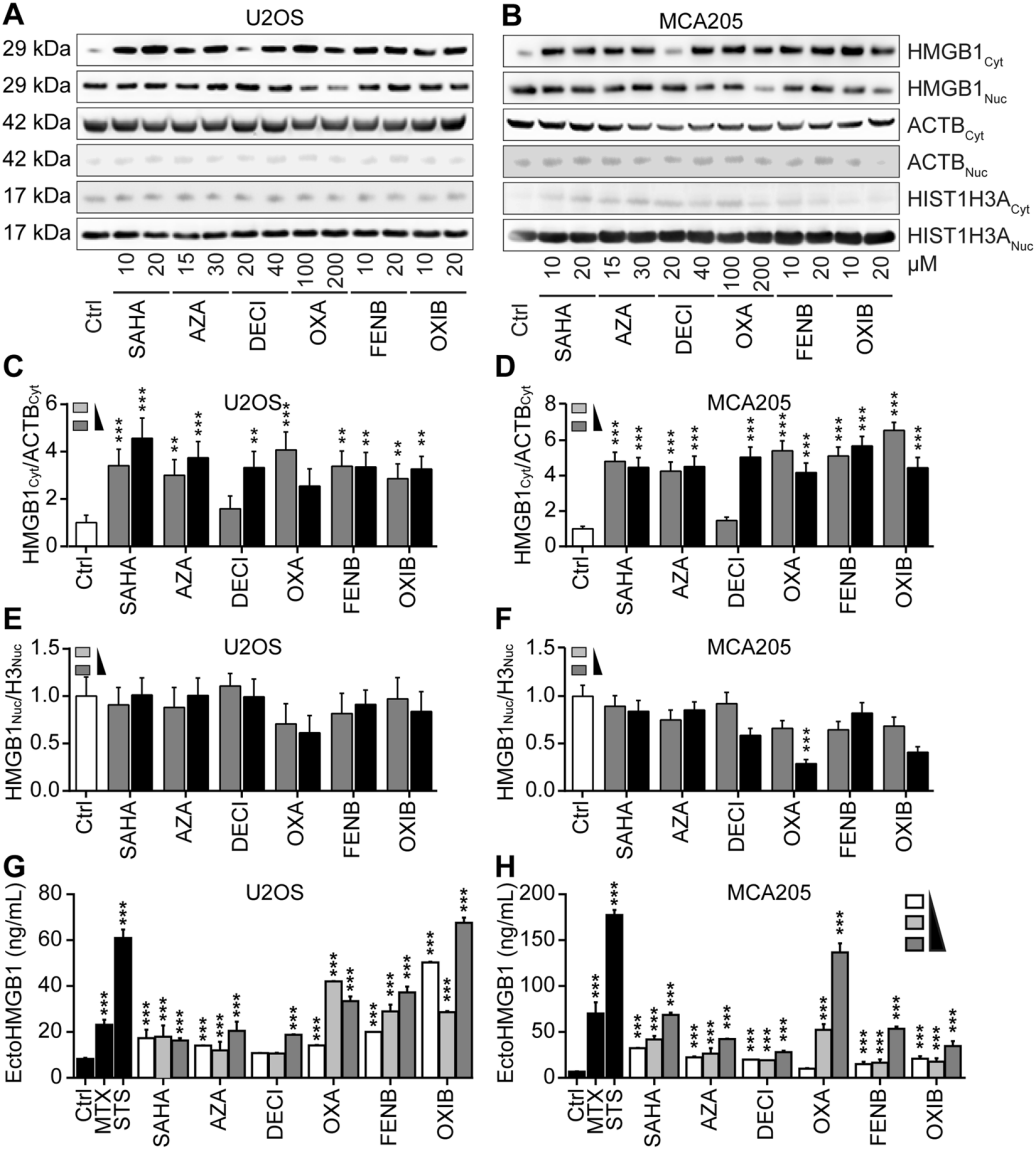
Biochemical detection of HMGB1 release. Human osteosarcoma U2OS cells or murine fibrosarcoma MCA205 cells were maintained in control conditions (Ctrl), suberoylanilide hydroxamic acid (SAHA), azacitidine (AZA), decitabine (DECI), oxaliplatin (OXA), fenbendazole (FENB) and oxibendazole (OXB), at indicated concentrations for 48 h, followed by subcellular fractionation and detection of nuclear and cytoplasmic HMGB1 expression by western blotting. Beta-actin and Histone H3 were used as loading controls of cytoplasmic and nuclear proteins respectively. Representative immunoblots (**A,B**) and densitometry data (**C–F**) are depicted. Densitometry data are represented as mean ± SEM of three independent experiments. (**G,H**) Cells were treated with staurosporine (STS) at 1 µM; mitoxantrone (MTX) at 3 µM; suberoylanilide hydroxamic acid (SAHA), fenbendazole (FENB) and oxibendazole (OXB) at 5, 10, 20 µM, azacitidine (AZA) at 7.5, 15, 30 µM, decitabine (DECI) at 10, 20, 40 µM; and oxaliplatin (OXA) at 50, 100, 200 µM for 48 h, followed by the assessment of HMGB1 (EctoHMGB1) release into cell culture supernatants by means of an HMGB1-specific ELISA. Data are reported as means ± SEM (n = 3; ***P < 0.001, two-tailed Student’s t test, compared to Ctrl cells) Additional data on MTX and STS-induced HMGB1 release can be found in Fig. S6).

**Figure 7:**
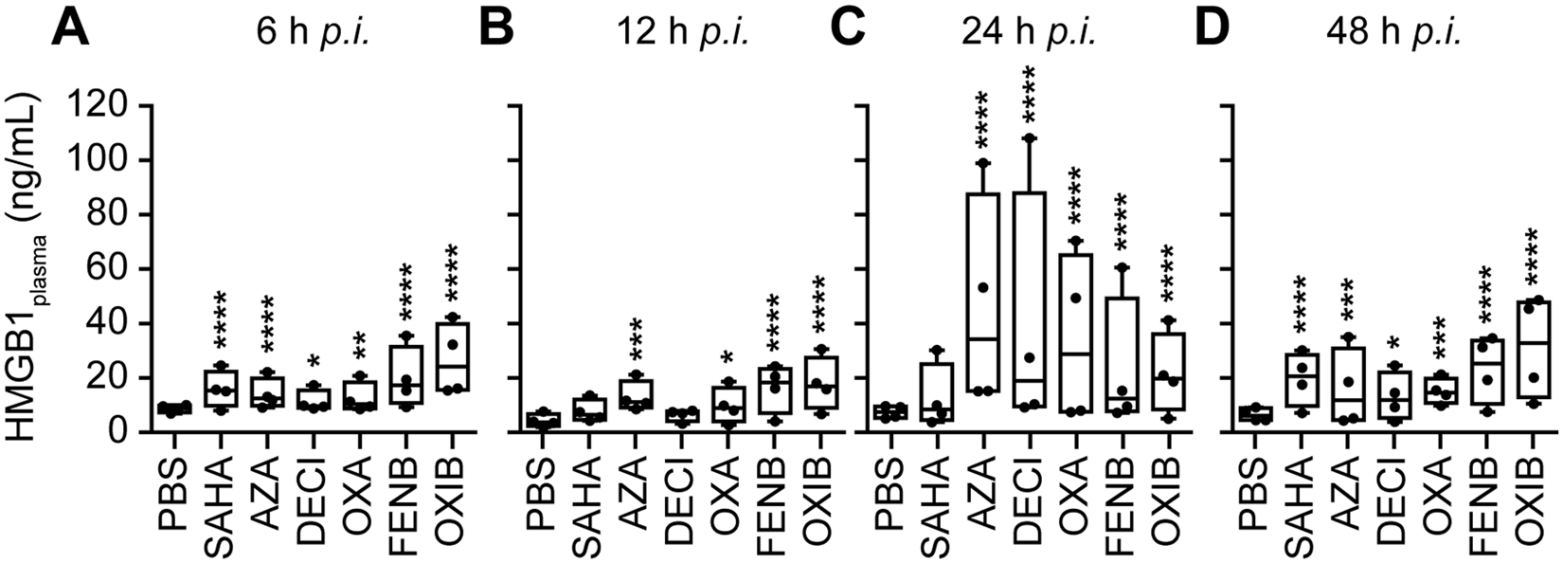
HMGB1 increases in mice plasma post IP injection of HMGB1 releasing agents. Female C57BL/6 mice (4 mice/group) were intraperitoneally administrated with suberoylanilide hydroxamic acid (SAHA) (100 mg/Kg), azacitidine (AZA) (50 mg/Kg), decitabine (DECI) (100 mg/Kg), oxaliplatin (OXA) (10 mg/Kg), fenbendazole (FENB) (200 mg/Kg) and oxibendazole (OXIB) (200 mg/Kg). Blood samples were collected at the indicated time points *post injection* (*p*.*i*.) and plasma was prepared as described in material and methods. Plasma HMGB1 levels were measured by means of a HMGB1-specific ELISA kit according to the manufacture protocol. Boxplots report the lower and upper quartile plus the median value. *p < 0.05, **p < 0.01, ***p < 0.001, ****p < 0.0001 (two-way ANOVA analysis).

In the next step, we determined whether the aforementioned compounds may also release a GFP-HMGB1 fusion protein (without SBP), as well as the endogenous HMGB1 protein (without GFP). For this, U2OS cells that were stably transduced with GFP-HMGB1 were exposed to SAHA, azacitidine, decitabine, oxaliplatin, fenbendazole and oxibendazole, and the increase in the cytoplasmic GFP-HMGB1 signal was confirmed (Fig. 5A–C). Alternatively, parental U2OS cells (devoid of GFP) were cultured with SAHA, azacitidine, decitabine, oxaliplatin, fenbendazole and oxibendazole, then fixed, permeabilized and subjected to immunofluorescence staining of HMGB1, again confirming its nucleo-cytoplasmic translocation (Fig. 5D–F). This result was further corroborated using subcellular fractionation to separate the nuclei from the cytoplasm, followed by immunoblot detection of HMGB1 (Fig. 6A–F). Moreover, an HMGB1-specific enzyme-linked immunosorbent assay (ELISA) revealed a significant, dose-dependent release of HMGB1 into the supernatant of the cells cultured in the presence of SAHA, azacitidine, decitabine, oxaliplatin, fenbendazole or oxibendazole. This result was obtained with U2OS cells (Fig. 6G), as well as with mouse methylcholantrene-induced fibrosarcoma MCA205 cells (Fig. 6H). Moreover, intraperitoneal injection of SAHA, azacitidine, decitabine, oxaliplatin, fenbendazole and oxibendazole into mice caused a time-dependent increase in the plasma concentration of ELISA-detectable HMGB1 (Fig. 7). Altogether, these results confirm that SAHA, azacitidine, decitabine, oxaliplatin, fenbendazole and oxibendazole act as potent inducers of HMGB1 release.

### Mode of action of HMGB1 release

**Figure 8:**
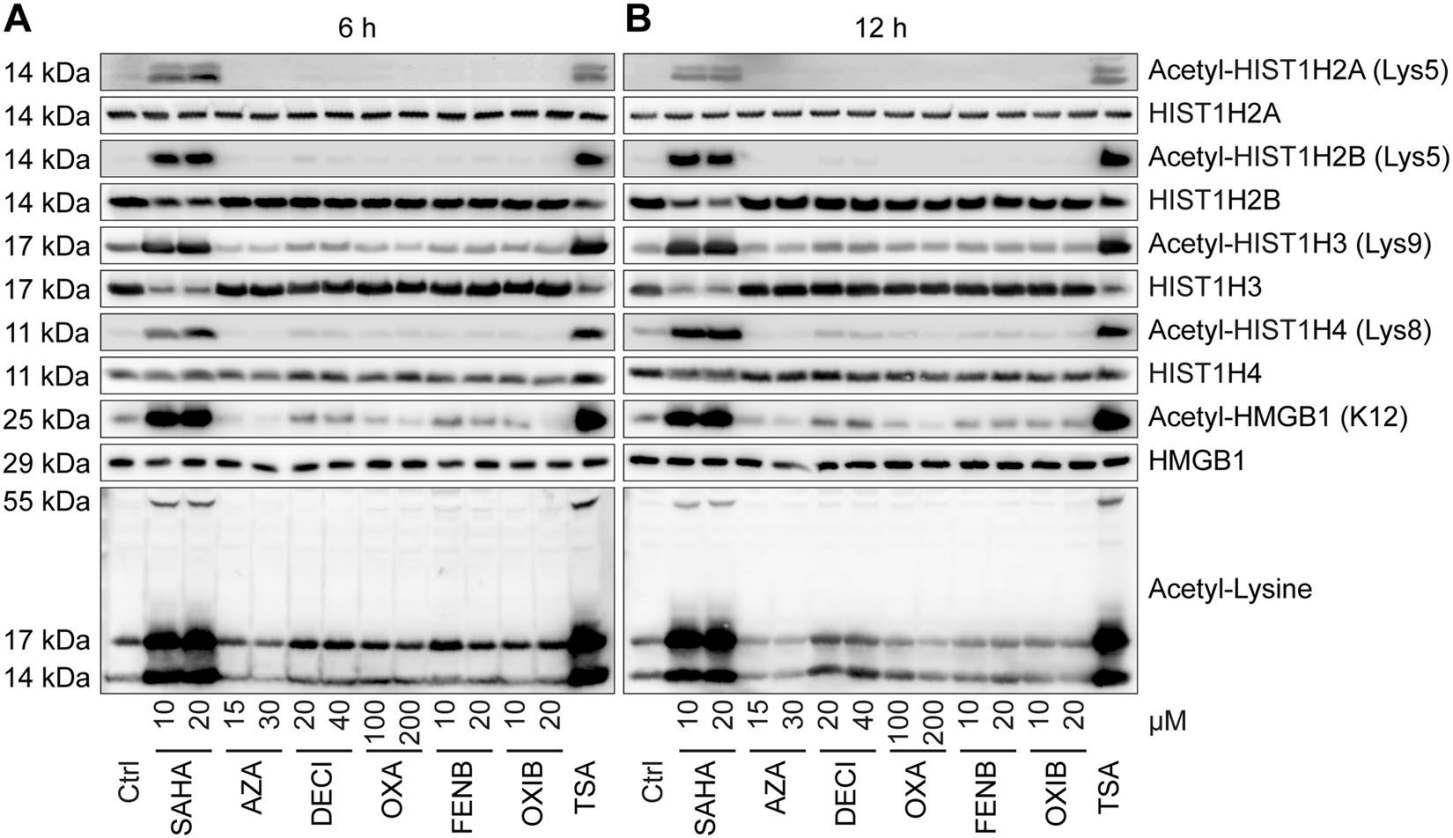
Effects of SAHA, Azacitidine, Decitabine, Oxaliplatin, Fenbendazole and Oxibendazole on the acetylation of histones and HMGB1. U2OS cells were maintained in control conditions (Ctrl), or were treated with suberoylanilide hydroxamic acid (SAHA), azacitidine (AZA), decitabine (DECI), oxaliplatin (OXA), fenbendazole (FENB) and oxibendazole (OXIB) at the indicated concentrations for 6 (**A**) or 12 (**B**) h, followed by total protein extraction and detection of acetylated/deacetylated histones and HMGB1 by western blotting.

**Figure 9:**
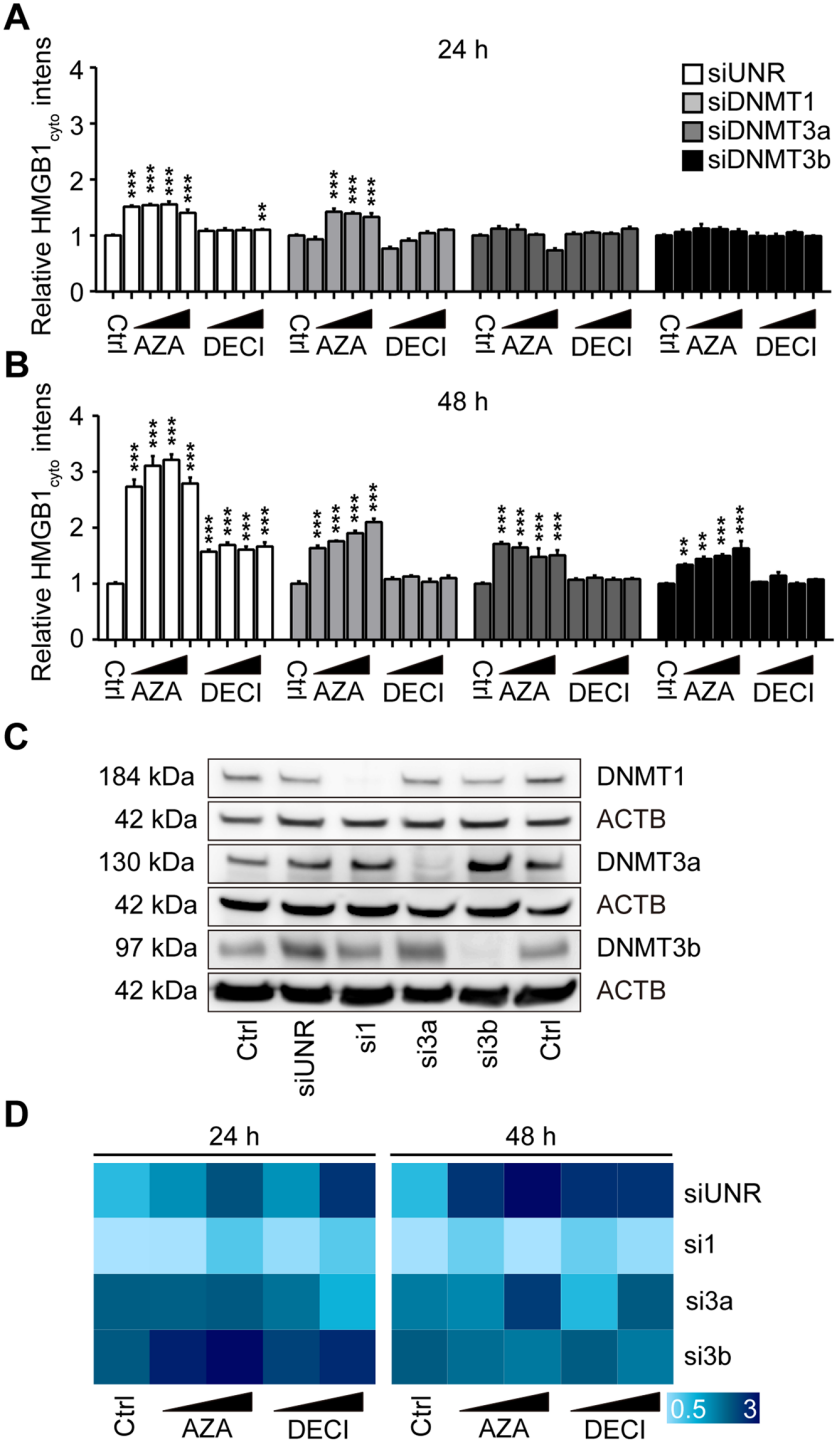
Effect of DNMT knockdown on azacitidine- and decitabine-induced HMGB1 release. U2OS cells expressing GFP-HMGB1 were transfected with small interfering RNAs (siRNA) targeting DNA methyl transferases 1 (DNMT1), 3a (DNMT3a) and 3b (DNMT3b). Forty-eight hours post transfection, the cells were either cultured in control conditions or exposed to azacitidine (AZA; 15, 30 µM) or decitabine (DEZI; 15, 30 µM) for additional 24 or 48 hours followed by assessment of cytoplasmic GFP-HMGB1 fluorescence (**A**,**B**). Cytoplasmic GFP intensity was quantified and normalized to untreated controls of respective siRNAs transfection. Data are shown as means ± SEM (n = 4; *P < 0.05, **P < 0.01, and ***P < 0.001, two-tailed Student’s t test, compared to control wells). Knockdown efficacy was validated by western blot (**C**). SiRNAs targeting DNMT1, DNMT3a and DNMT3b were utilized in U2OS GFP-HMGB1 expressing cells following the same protocol as described above. Cells were treated with AZA (15, 30 µM) and DECI (15, 30 µM) for 24 or 48 h and normalized cytoplasmic GFP intensity was reported as heat map (**D**).

We investigated possible communalities in the mode of action of SAHA, azacitidine, decitabine, oxaliplatin, fenbendazole and oxibendazole. Previously, histone deacetylase inhibitors such as trichostatin A (TSA) have been shown to stimulate the hyperacetylation of HMGB1 as well as histones which facilitates the release of HMGB1 from chromatin^2,5,29^. Nonetheless, only SAHA was able to cause an increase in histone acetylation, while none of the other agents did so (Fig. 8). One physiological cellular state in which HMGB1 is released from the nucleus is mitosis where HMGB1-SBP-GFP can be found in a cytoplasmic location, provided that biotin has been added to the cells to unlink reporter and hook (Fig. S4, Videos S3 and S4). However, only the histone deacetylase (HDAC) inhibitors trichostatin A (TSA, as a positive control), SAHA and anthelmintic agents fenbendazole and oxibendazole caused a major blockade of the cell cycle in mitosis, contrasting with azacitidine, decitabine as well as oxaliplatin, which arrested the cells in the S-phase (Fig. S5). Hence, the drugs characterized here do not have a common cell cycle-blocking activity that would explain their effects on HMGB1 nuclear release. We also investigated the possibility that azacitidine and decitabine would act by inhibiting their known pharmacological targets (which are the DNA methyl transferases 1, 3a and 3b; DNMT1, DNMT3a, DNMT3b)^30,31^ or rather through off-target effects. The knockdown of DNMT3a or DNMT3b, was sufficient to induce the nucleo-cytoplasmic translocation of GFP-HMGB1, and this effect was not further enhanced by azacitidine and decitabine (Fig. 9). These finding plead in favor of an on-target effect of azacitidine and decitabine.

### Concluding remarks

In the present paper, we developed a robust assay for identifying pharmacological agents that cause the nuclear release of HMGB1. Supporting the specificity of the screening system that we developed, we identified several drugs that fell into two categories (epigenetic modifiers, microtubule inhibitors) as *bona fide* inducers of HMGB1 translocation. Indeed, these agents as well as some classical cytotoxicants (mitoxantrone, oxaliplatin) were highly efficient in stimulating HMGB1 release, both *in vitro*, on cultured human or mouse cells, as well as *in vivo*, in mice. Multiple distinct technologies to detect HMGB1 release yielded coherent results, as documented for the RUSH assay, the subcellular localization of an GFP-HMGB1 fusion protein (independent from RUSH), the immunofluorescence detection of endogenous HMGB1 protein, immunoblot determinations after subcellular fractionation, as well as ELISA-based detection of extracellular HMGB1. Importantly, the mode of action of these agents with respect to HMGB1 release are probably heterogeneous, because they were not linked to a common mechanism of protein hyperacetylation or similar cell cycle effects. Interestingly, DNA hypomethylating agents induced nuclear HMGB1 exodus via on-target effects, suggesting that the methylation status of DNA determines chromatin interaction with HMGB1. However, further studies are necessary to understand such an effect, given that so far HMGB1 binding to chromatin only has been related to histone H3K9 dimethylation, not to DNA methylation^32^. Moreover, an increase (not a decrease) of the methylation of HMGB1 itself has been linked to its release^33^.

In the present study microtubule targeting agents with both stabilizing and de-stabilizing activity have been identified to release HMGB1 from the nucleus. Thus, nocodazole as well as the antihelmintic agents albendazole, fenbendazole, flubendazole, mebendazole, oxibendazole, that exert their mode of action by binding to tubulin to inhibit the polymerization of microtubules induce the nuclear exodus of HMGB1. Similarly, taxanes, such as paclitaxel and docetaxel, which interfere with microtubule depolymerization thereby inhibiting their dynamics, cause HMGB1 release. Binding of HMGB1 to tubulin through its central B domain has been suggested based on *in situ* experiments^34^, yet information on direct interaction remains scarce and further in depth analysis is needed. In the present study we excluded toxic microtubule inhibitors from *in vivo* validations.

In conclusion, we have identified several classes of agents as potent inducers of the nucleo-cytoplasmic relocation and subsequent cellular release of HMGB1. It will be interesting to learn whether such effects may contribute to the immunostimulatory effects of drugs that are used to treat malignant disease or worm infection.

## Materials and Methods

### Cell culture, reagents and antibodies

Human osteosarcoma U2OS cells and murine fibrosarcoma MCA205 cells were cultured in Dulbecco’s Modified Eagle Medium (DMEM) supplemented with 10% fetal bovine serum (FBS), 100 U/mL penicillin, and 100 μg/mL streptomycin in a humidified atmosphere containing 5% CO2 at 37 °C^35,36^. All cell culture media and supplements were from Gibco-Invitrogen (Carlsbad, CA, USA) and all plastic materials came from Corning (Corning, NY, USA).

The Prestwick Chemical Library was obtained from Prestwick Chemical (Illkirch, France). Small molecules FDA-approved as anticancer agents included in the custom library, mitoxantrone (MTX), vorinostat (SAHA), azacitidine, decitabine, oxaliplatin, fenbendazole, oxibendazole, butyrate, isobutyrate, biotin, avidin, Trichostatin A (TSA) were purchased from Sigma-Aldrich (St Louis, MI, USA). Geneticin (G418) and hygromycin (Hygro) were purchased from Invivogen (Eugene, OR, USA).

Primary antibodies against streptavidin (mouse), DNMT1 (mouse), DNMT3a (mouse), DNMT3b (mouse) were obtained from Santa Cruz Biotechnology (Dallas, TX, USA) HMGB1 (rabbit), beta-actin-HRP were obtained from Abcam (Cambridge, UK), acetyl-HMGB1 (K12) (rabbit) was from Elabscience Biotechnology Inc, (Wuhan, China) and acetylated-lysine (rabbit) and acetyl-histone antibody sampler kit were from Cell Signaling Technology (Danvers, MA, USA). Secondary antibodies were from SouthernBiotech (Birmingham, AL, USA).

### Stable plasmid transduction

Lentiviral particles were produced using the ViraPower™ Lentiviral Packaging Mix (Thermo Fisher Scientific, Waltham, MA) according to the manufacturers’ protocol. U2OS cells were infected with the RUSH hook streptavidin-NLS3-bearing lentiviral particles and following the transduced cells were selected for 2 weeks under the continuous presence of G418 (0.5 mg/mL). U2OS-NLS3 hook clones were generated by single cell-sorting on a FACS ARIA III cytofluorometer (Becton Dickinson, San José, CA, USA), and the most potent U2OS-NLS3 hook clone was selected by immunofluorescence staining.

For the expression of HMGB1 reporter, U2OS NLS3 hook cells were further transfected with RUSH reporter-HMGB1-SBP-GFP-bearing (HMGB1 accession number: NM_002128.4) lentiviral particles by means of ViraPower™ lentiviral expression system. Transfected cells were cultured in the presence of hygromycin (0.5 mg/mL) for 2 weeks, and U2OS-Str-NLS3 HMGB1-SBP-GFP positive clones were selected by a FACS ARIA III cytofluorometer.

U2OS GFP-HMGB1 stable expressing cells were generated by means of LentiBrite™ GFP-HMGB1 Lentiviral Biosensor (Merck Millipore, Billerica, MA, USA) following the manufacturer’s instructions. Following cells were cloned and the most potent clones were selected by visual inspection.

### High-throughput screen for HMGB1-releasing drugs

U2OS cells stably co-expressing streptavidin-NLS3, HMGB1-SBP-GFP (1.5 × 10^3^ cells per well) were seeded into black 384-well imaging plates (GreinerBioOne, Kremsmünster, Austria) and let adapt for 24 hours. Following the compounds from the Prestwick Chemical Library and the Custom Library were added in the presence or the absence of biotin (40 µM). Forty-eight hours later, cells were stained with 2 μM CellTracker™ Orange CMTMR (Thermo Fisher Scientific) for another hour. Thereafter cells were rinsed with PBS, and fixed with 4% PFA containing 2 µg/mL Hoechst 33342 overnight at 4 °C. After 3 additional washing steps cells were superseded with 50 μL PBS and subjected to automated image acquisition and subsequent image analysis. For automated fluorescence microscopy, a robot-assisted Molecular Devices IXM XL BioImager (Molecular Devices, Sunnyvale, CA, USA) equipped with a Sola light source (Lumencor, Beaverton, OR, USA), adequate excitation and emission filters (Semrock) a 16-bit monochrome sCMOS PCO.edge 5.5 camera (PCO, Kelheim, Germany) and a 20 X PlanAPO objective (Nikon, Tokyo, Japan) was used to acquire at least 4 view fields of each well. Following images were processed with the MetaXpress image analysis software (Molecular Devices). Images were segmented using the built-in custom module editor to identify nuclei (based on Hoechst 33342 signal), and cytoplasmic regions (based on CellTracker™ Orange CMTMR fluorescence), allowing the quantification of nuclear/cytoplasmic HMGB1-GFP intensity. Data were mined and statistically evaluated using the freely available software R (https://www.r-project.org). Data were intra-plate normalized by evaluating the ratio to plate means and inter-plate normalized by calculating Z-scores.

$$Z=\frac{X{\rm{i}}-mean(Xall)}{std(Xall)}$$

### Flow cytometric analysis of cell cycle

U2OS cells were synchronized in G1 phase by double thymidine blockade and washed twice before treatment with SAHA, Azacitidine, Decitabine, Oxaliplatin, Fenbendazole and Oxibendazole for additional 24 h. Then, cells were harvested, washed twice with ice-cold PBS, resuspended in 1 mL 75% ethanol (−20 °C) and fixed at 4 °C overnight. Fixed cells were washed with PBS. Then, RNase A was added (50 µg/mL) for 5 min at room temperature, and stained with PI (50 µg/mL) in the dark at 4 °C for additional 30 min before cell cycle profiling on a FACS Fortessa flow cytometer (BD Bioscience, San Jose, CA).

### Western blotting

Cells were washed twice with ice-cold PBS and lysed in RIPA lysis buffer containing protease inhibitor cocktail (Roche, Basel, Switzerland). Alternatively, cytoplasmic and nuclear proteins of U2OS or MCA205 cells were separated and prepared by NE-PER™ Nuclear and Cytoplasmic Extraction Kit (Thermo Fisher Scientific) according to the manufacturer’s protocol. The concentration of total protein content was measured using a BCA protein assay kit (Thermo Fisher Scientific). Twenty µg of protein was resolved by SDS-PAGE gel (Invitrogen) and transferred to PVDF membranes (Merck Millipore). Membranes were blocked in TBS containing 0.01% Tween-20 and 5% non-fat dry milk for 1 h, and incubated with primary antibody overnight at 4 °C on a rocking shaker. Membranes were then washed five times with TBST for 10 min each, followed by incubation with secondary antibody for 2 h. Following membranes were washed with TBST and peroxidase activity was visualized with Amersham ECL Prime Western Blotting Detection Reagent (GE Healthcare, Little Chalfont, UK) and an ImageQuant LAS4000 (GE Healthcare). Densitometry was conducted using the ImageQuantTL software (GE Healthcare).

### Immunofluorescence

Cells were seeded into 96 or 384 wells black microplates and let adhere for 24 hours. Following treatment cells were washed twice with PBS (room temperature) fixed with 4% PFA which containing 2 µg/mL Hoechst 33342 for 10 min, washed twice with PBS and quenched for 5 min with quenching solution (2.67 g NH_4_Cl in 1 L PBS, pH 7.4). Cells were permeabilized with 0.1% Triton-X100 for 10 min, and rinsed 3 times with PBS and were then blocked with 1% BSA in PBST for 30 min. Cells were incubated overnight at 4 °C with primary antibodies, rinsed 3 times with PBS, and then incubated with secondary antibodies for 1 h at room temperature. After 3 additional washing steps, 100 µL (96 wells plate) or 50 µL (384 wells plate) of PBS were added. The plates were subjected to automated image acquisition and subsequent image analysis as described above.

### Determination of the concentration of HMGB1 in cell culture supernatant and murine plasma

The concentration of extracellular HMGB1 in cell culture supernatant and murine blood plasma was quantified, by mean of the HMGB1 ELISA KIT (Tecan, Switzerland) following the manufactures’ instructions.

### Determination of HMGB1 release *in vivo*

All animal experiments were used in compliance with the French and European rules concerning animal experimentation (EU 2010/63) and were approved by the Ethical Committee of the Gustave Roussy Cancer Campus (Villejuif, France) (#4333-2016030212117545). C57BL/6 mice (Envigo, Cambridgeshire, UK), 6 weeks old females, were housed in a 12 h light/dark cycles with a temperature-controlled environment. PBS, SAHA (100 mg/Kg), azacitidine (50 mg/Kg), decitabine (100 mg/Kg), oxaliplatin (10 mg/Kg), fenbendazole (200 mg/Kg) or oxibendazole (200 mg/Kg) was intraperitoneally injected. Mice were divided into 4 groups regarding 4 time points (6, 12, 24, 48 h post injection). Blood samples were collected at the indicated time points according to standard methods before mice were euthanized by cervical dislocation. Plasma samples were prepared by centrifugation and HMGB1 concentration was determined by ELISA (Tecan, Switzerland).

### siRNA transfection

SMARTpool siGENOME siRNAs for silencing the expression of DNMT1, DNMT3a, DNMT3b as well as *non target* control siRNAs were obtained from GE Dharmacon (Chicago, Il, USA). All transfection procedures strictly followed the siRNA Transfection Protocol of DharmaFECT™ Transfection Reagents using a final siRNA concentration of 25 nM, and of 0.05 uL/well for 384-well plates of DharmaFECT™ Transfection Reagents.

### Statistical analyses

Unless otherwise specified, data are reported as mean ± SEM. Statistical significance was analyzed using the Student’s-test. Differences in treated and control cells were considered to be significant if ^*^p < 0.05, ^**^p < 0.01 ^***^p < 0.001.

## Electronic supplementary material

Supplementary Video 1

Supplementary Video 2

Supplementary Video 3

Supplementary Video 4

Supplemental Information
