# Supplementary material for: Identification of pharmacological agents that induce HMGB1 release
Source: Sci Rep. 2017 Nov 2;7:14915. doi: 10.1038/s41598-017-14848-1 (PMC5668281; doi:10.1038/s41598-017-14848-1)
Supplement: Supplementary file 5 — Supplemental Information [file 41598_2017_14848_MOESM5_ESM.doc]

**Supplemental files**

**Identification of pharmacological agents that induce HMGB1 release**

**Peng Liu**^1-6^**, Liwei Zhao**^1-6^**, Friedemann Loos**^1-6^**, Kristina Iribarren**^1-6^**, Sylvie Lachkar**^1-6^**, Heng Zhou**^1-6^**, Lígia C Gomes-da-Silva**^1-6^, **Guo Chen**^1-6^, **Lucillia Bezu**^1-6^**, Gaelle Boncompain**^7^**, Franck Perez**^7^**, Laurence Zitvogel**^1,8,9,10*^**, Oliver Kepp**^1-6*^**, and Guido Kroemer**^2-6,11,12*^

^1^Faculty of Medicine, University of Paris Sud, Kremlin-Bicêtre, France; ^2^Cell Biology and Metabolomics Platforms, Gustave Roussy Cancer Campus, Villejuif, France; ^3^Equipe 11 labellisée Ligue Nationale contre le Cancer, Centre de Recherche des Cordeliers; Paris, France; ^4^Institut National de la Santé et de la Recherche Médicale (INSERM), UMR1138, Equipe labellisée Ligue Nationale Contre le Cancer, Paris, France; ^5^Université Paris Descartes, Sorbonne Paris Cité, Paris, France; ^6^Université Pierre et Marie Curie, Paris, France; Institut Curie, PSL Research University, CNRS UMR144, Paris, France; ^8^Institut de Cancérologie Gustave Roussy Cancer Campus (GRCC), Villejuif, France ; ^9^INSERM, U1015, Villejuif, France; ^10^Center of Clinical Investigations CIC1428, Villejuif, France; ^11^Pôle de Biologie, Hôpital Européen Georges Pompidou, AP-HP; Paris, France; ^12^Department of Women's and Children's Health, Karolinska University Hospital, Stockholm, Sweden.

* LZ, OK and GK are senior co-authors of this paper.

Correspondence to: **Dr. Guido Kroemer** [**kroemer@orange.fr**](mailto:kroemer@orange.fr)

**Dr. Oliver Kepp** [captain.olsen@gmail.com](mailto:captain.olsen@gmail.com)

**Supplemental figures and legends**

**
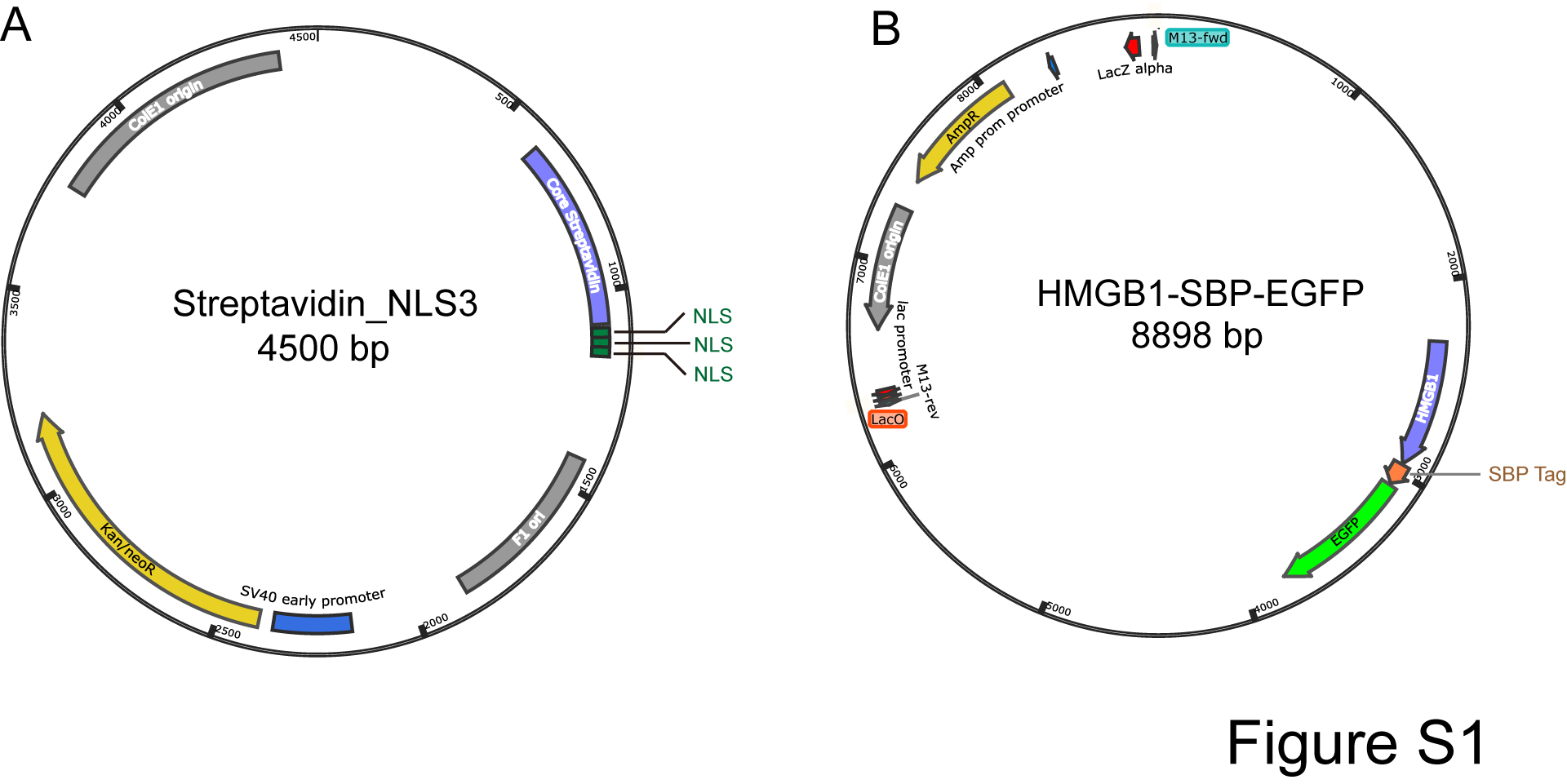
**

**Figure S1. Plasmid maps of pCDH_Streptarvidin-NLS3 and pCD-HMGB1-SBP-GFP.**

**
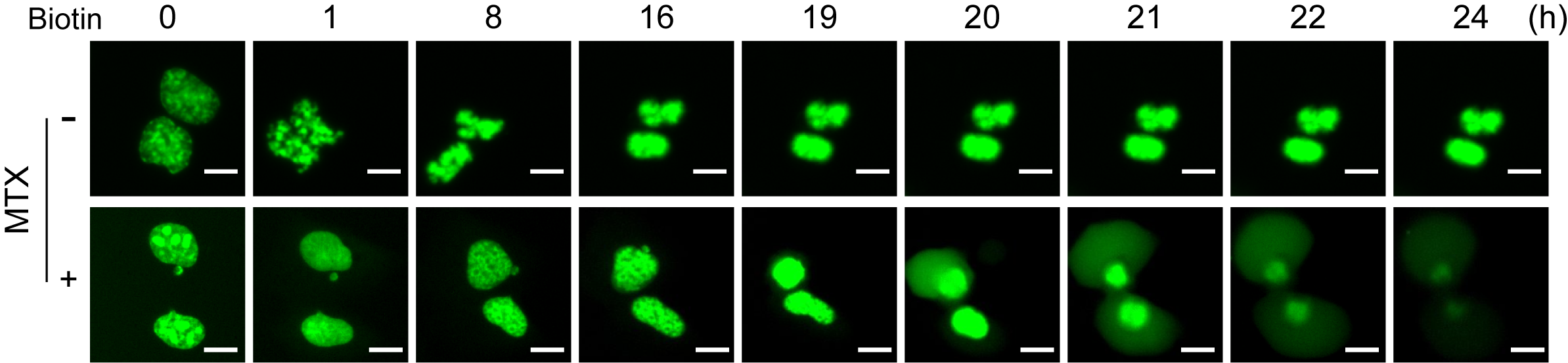
**

**Figure S2. Living cell video showed** **MTX induce HMGB1 release only in the presence of biotin.** U2OS-SBP-HMGB1, Streptavidin-NLS3 co-expressing cells were seeded in 96-well plate in the absence of biotin, 24h later medium was replaced with fresh medium containing 2 µM mitoxantrone (MTX) either in the absence or presence of biotin. Live cell imaging was launched immediately after. Representative images focusing on certain cells are reported, scale bar = 10 µm.


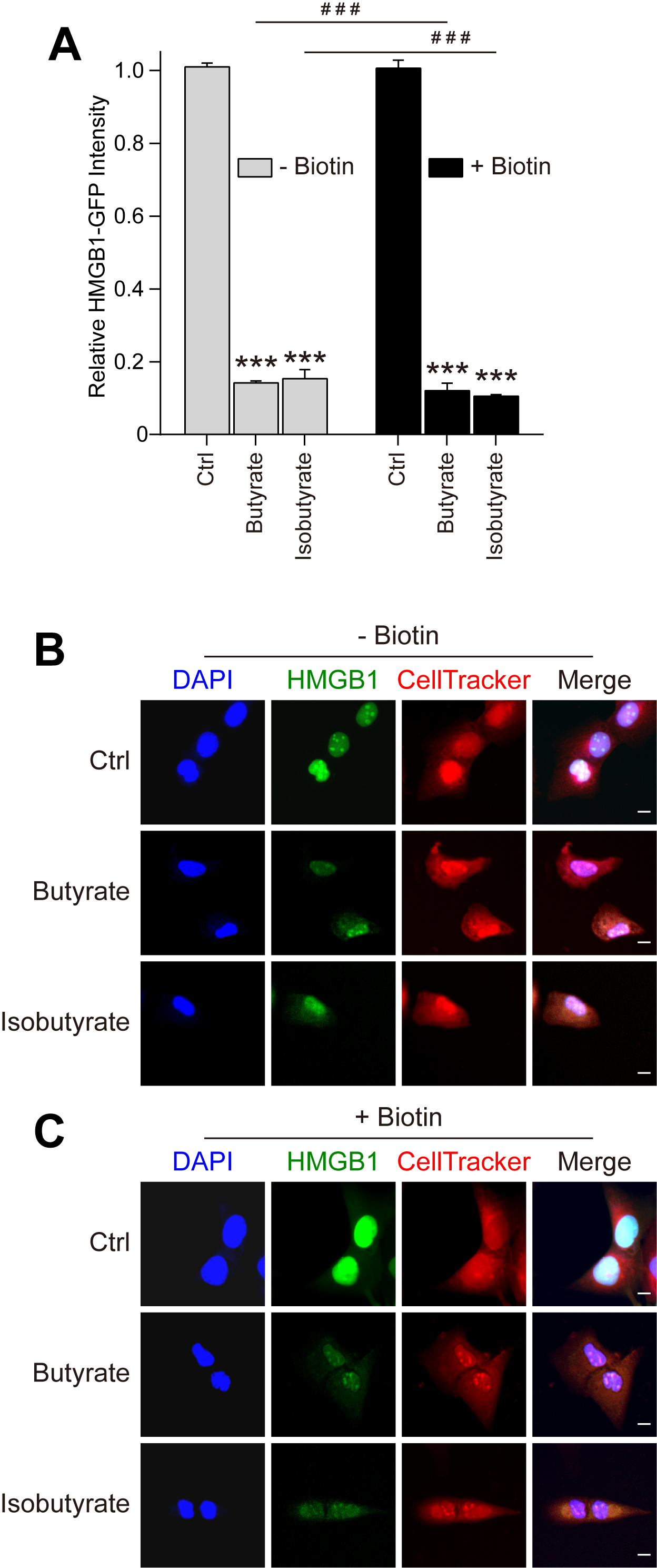
**Figure S3. Fluorescence quenching reagents decrease GFP intensity independent of biotin.** U2OS-SBP-HMGB1, Streptavidin-NLS3 co-expressing cells were co-cultured with fatty acids butyrate or isobutyrate either in the absence (B) or presence (C) of biotin for 24 h, before assessment of GFP intensity. Data was normalized to untreated condition and is shown as means ± SEM (n = 4; ns not significant, ***P < 0.001, two-tailed Student’s t test).


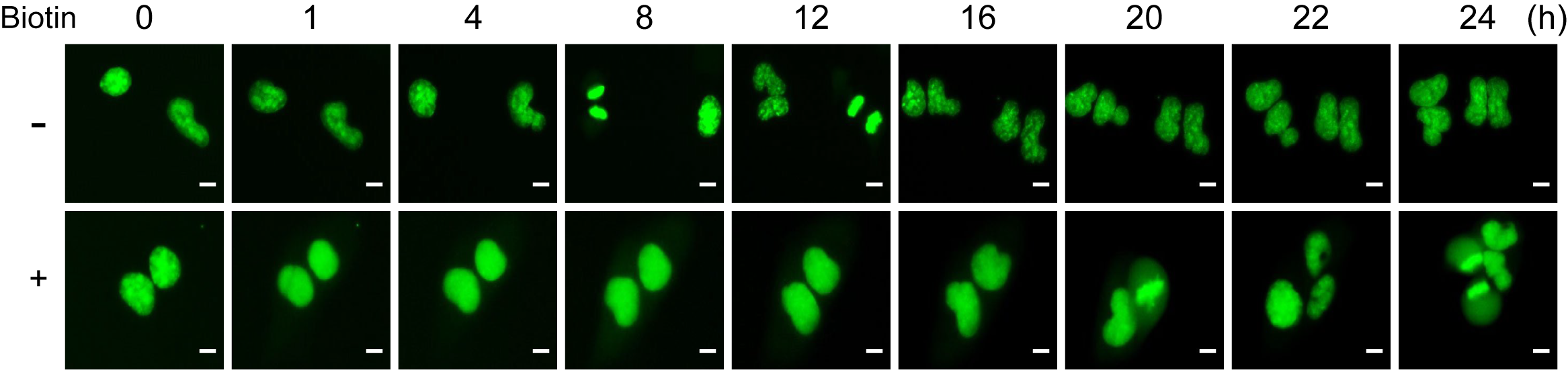


**Figure S4. HMGB1 releases to the cytoplasm during mitosis only in the presence of biotin.** U2OS-SBP-HMGB1, Streptavidin-NLS3 co-expressing cells were seed in 96-well plate in the absence of biotin, 24 h later medium was replaced with fresh medium containing or not biotin. Live cell imaging was launched immediately after. Representative images focusing on certain cells are reported, scale bar = 10 µm.

**
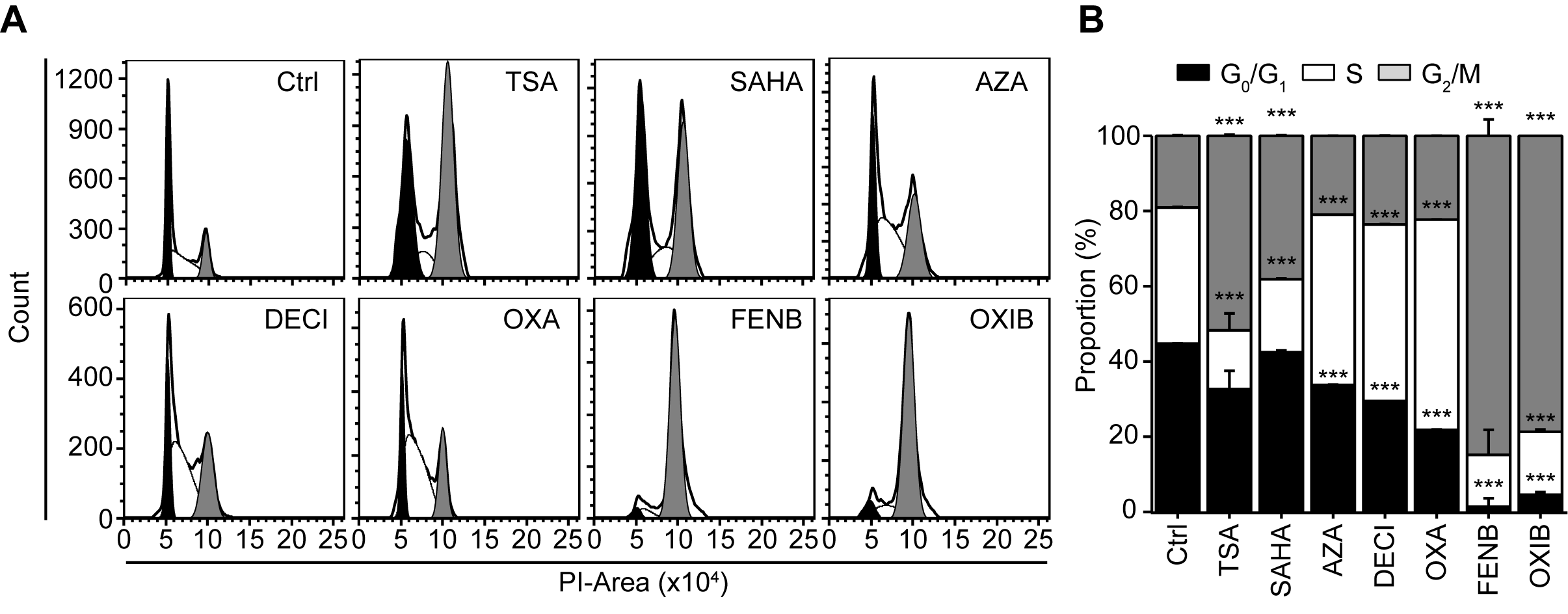
**

**Figure S5. Effects of SAHA, Azacitidine, Decitabine, Oxaliplatin, Fenbendazole and Oxibendazole on cell cycle progression.** U2OS cells were synchronized to G1 phase by double thymidine blockade and washed before treatment with trichostatin A (TSA), suberoylanilide hydroxamic acid (SAHA), azacitidine (AZA), decitabine (DECI), oxaliplatin (OXA), fenbendazole (FENB) and oxibendazole (OXIB) for 24 h. Then cells were fixed with 75% ethanol followed by PI staining for FACS. Cell cycle analysis was performed by FlowJo and data is reported as means ± SEM (n = 3; *P < 0.05, **P < 0.01, ***P < 0.001, two-tailed Student’s t test).


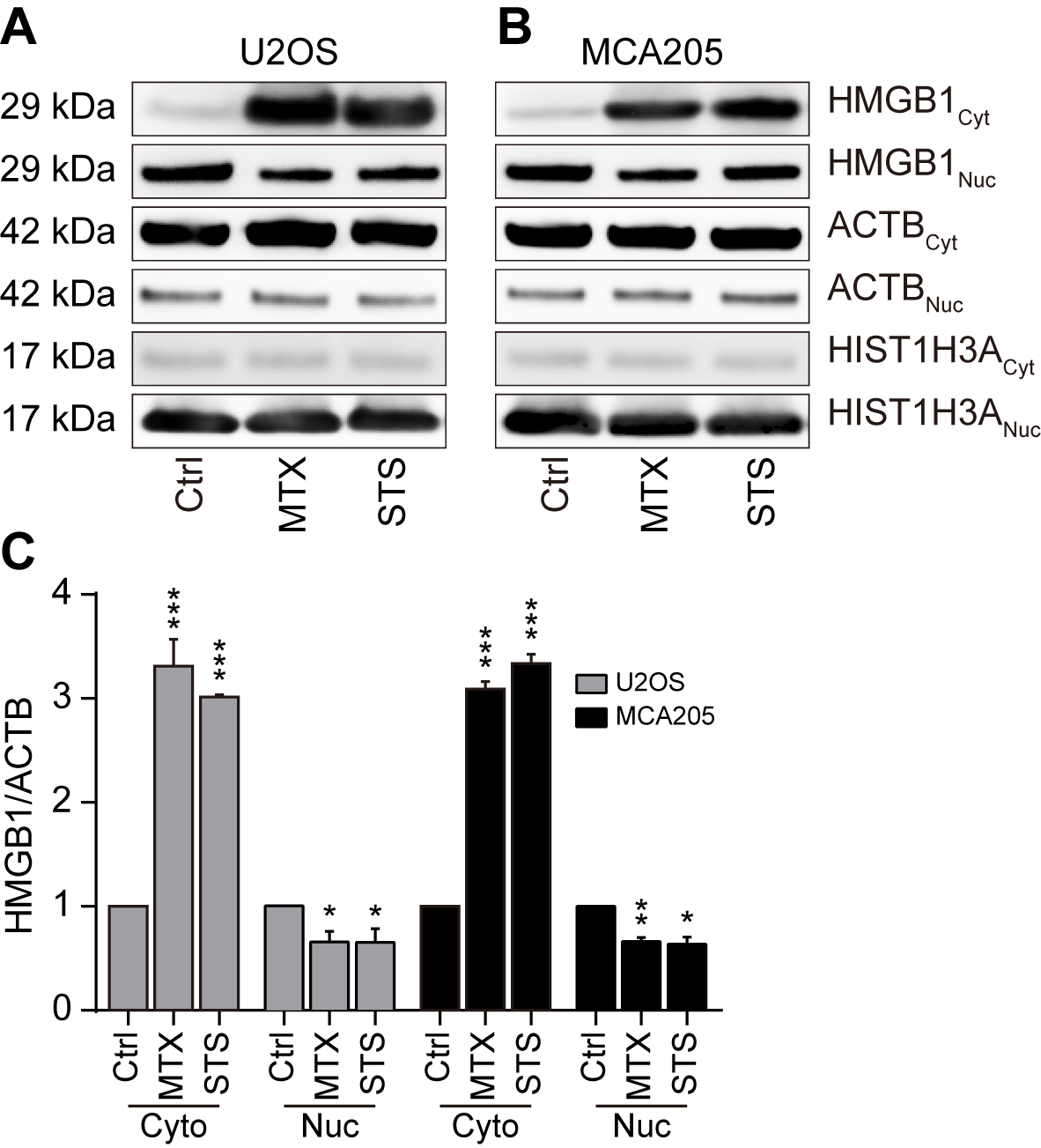


**Figure S6. Detection of MTX and STS induced HMGB1 release by western blotting.** Human osteosarcoma U2OS cells or murine fibrosarcoma MCA205 cells were maintained in control conditions (Ctrl), 3 µM MTX or 1 µM STS for 48 h, followed by subcellular fractionation and detection of nuclear and cytoplasmic HMGB1 expression by western blotting. Beta-actin and Histone H3 were used as loading controls of cytoplasmic and nuclear proteins respectively. Representative immunoblots (A, B) and densitometry data (C) are depicted. Densitometry data are represented as mean ± SEM of three independent experiments, *P < 0.05, **P < 0.01, ***P < 0.001, two-tailed Student’s t test, as compared to Ctrl cells.

**Legends to supplemental videos**

**Video S1. Failure to release HMGB1-SBP-GFP in response to MTX in the absence of biotin.** U2OS-SBP-HMGB1, Streptavidin-NLS3 co-expressing cells were seeded in 96-well plate in the absence of biotin, 24 h later medium was replaced with fresh medium containing 2 µM mitoxantrone but no biotin. Live cell imaging was launched immediately after. Images were acquired every 90 seconds for the first 30 min using an automated bioimager equipped with environmental control by means of a 20 X PlanApo objective. Following images were taken every 20 min for a total of 24 hours.

**Video S2. Release of HMGB1-SBP-GFP in response to MTX in the presence of biotin.** U2OS-SBP-HMGB1, Streptavidin-NLS3 co-expressing cells were seeded as for video S1, twenty-four hours later medium was replaced with fresh medium containing 2 µM mitoxantrone and 40 µM biotin. Live cell imaging was conducted as described before.

**Video S3. Failure of HMGB1-SBP-GFP release during mitosis in the absence of biotin.** U2OS-SBP-HMGB1 and Streptavidin-NLS3 co-expressing cells were seeded as for video S1, twenty-four hours later medium was replaced with fresh medium without biotin. Live cell imaging was conducted as described before.

**Video S4. HMGB1-SBP-GFP release (and re-localization) during mitosis in the presence of biotin.** U2OS-SBP-HMGB1, Streptavidin-NLS3 co-expressing cells were seeded as for video S1, twenty-four hours later medium was replaced with fresh medium without biotin. Live cell imaging was conducted as described before.
